# A method for high throughput determination of viable bacteria cell counts in 96-well plates

**DOI:** 10.1186/1471-2180-12-259

**Published:** 2012-11-13

**Authors:** Ronen Hazan, Yok-Ai Que, Damien Maura, Laurence G Rahme

**Affiliations:** 1 Department of Surgery, Harvard Medical School and Massachusetts General Hospital, Boston, MA, 02114, USA; 2 Department of Microbiology and Immunobiology, Harvard Medical School, Boston, MA, 02114, USA; 3 Shriners Hospitals for Children Boston, Boston, MA, 02114, USA; 4 IYAR, The Israeli Institute for Advanced Research, Rehovot, Israel

**Keywords:** Bacterial-count, CFU, Persisters, High-Throughput, Screen

## Abstract

**Background:**

There are several methods for quantitating bacterial cells, each with advantages and disadvantages. The most common method is bacterial plating, which has the advantage of allowing live cell assessment through colony forming unit (CFU) counts but is not well suited for high throughput screening (HTS). On the other hand, spectrophotometry is adaptable to HTS applications but does not differentiate between dead and living bacteria and has low sensitivity.

**Results:**

Here, we report a bacterial cell counting method termed Start Growth Time (SGT) that allows rapid and serial quantification of the absolute or relative number of live cells in a bacterial culture in a high throughput manner. We combined the methodology of quantitative polymerase chain reaction (qPCR) calculations with a previously described qualitative method of bacterial growth determination to develop an improved quantitative method. We show that SGT detects only live bacteria and is sensitive enough to differentiate between 40 and 400 cells/mL. SGT is based on the re-growth time required by a growing cell culture to reach a threshold, and the notion that this time is proportional to the number of cells in the initial inoculum. We show several applications of SGT, including assessment of antibiotic effects on cell viability and determination of an antibiotic tolerant subpopulation fraction within a cell population. SGT results do not differ significantly from results obtained by CFU counts.

**Conclusion:**

SGT is a relatively quick, highly sensitive, reproducible and non-laborious method that can be used in HTS settings to longitudinally assess live cells in bacterial cell cultures.

## Background

Determination of bacterial cell number is among the most fundamental procedures in microbiology. Several methods are commonly used, each with its characteristic pros and cons (Table 1). The widely used gold standard method is Colonies Forming Units (CFU) counting on plates [1]. The CFU method has two noteworthy advantages, namely the capacity for counts of any number of bacteria using dilutions, if too many, or concentrations if too few. Second, only viable bacteria are counted with this method as the CFU method excludes dead bacteria and debris. The most important disadvantage of the CFU method is that clumps of bacteria cells can be miscounted as single colonies; the potential for counting clumps as single units is in fact reason the results are reported as CFU/mL rather than bacteria/mL. In addition, CFU results are usually obtained after 1–3 d, making the method not suitable for serial longitudinal studies. And since the CFU method is also relatively time-consuming and quite tedious, it has limitations for high throughput screening (HTS) studies.

**Table 1:** Bacteria quantification methods

**Method**	**Range of detection**	**Time to obtain results**	**Distinguishes live vs. dead**	**Persisters included in quantification**	**Applications**	**Equipment needed**	**Count affected by minor bacterial clumps**
CFU count	Unlimited	Days	Yes	Yes	Determination of absolute bacterial number	None	Yes
Absorbance	10^8^–10^10^ bacteria/mL	Immediate	No	No	Follow growth curves	Spectrophotometer or plate reader	No
Microscopy	Unlimited	Minutes	Yes, with staining	No	Determination of absolute bacterial number	Microscope	No
Flow cytometry	> ~5000	Minutes	Yes, with staining	Yes, if not below detection	Determination of absolute bacterial number	FACS	Yes
MBRT [2]	> ~10^7^	Hours	Yes	No (metabolically quiescent cells missed)	MIC and MAC determination	Spectrophotometer	No
**SGT**	Unlimited	Hours	Yes	Yes	HTS, persister Quantification	Plate reader	No

The other common method used to estimate bacterial load is reading optical density (OD) at 600 nm. The OD method can be performed automatically in a high throughput manner using a microtiter plate reader and is well suited for experiments requiring continuous growth curve analysis. However, this method does not distinguish live bacteria from dead bacteria or even particles. In addition, its sensitivity is usually limited to concentrations between 10^8^ and 10^10^ bacteria/mL.

Several other, but less common, methods for estimating bacterial concentration estimation have been described, including flow cytometry [3] and microscopy counting. These methods are sensitive and accurate, and investigators can distinguish between live and dead bacteria when appropriate dyes are employed. However, both are not suitable for HTS studies because are relatively time-consuming and quite tedious. Bacteria number can also be estimated based on various metabolic features, such as the methylene blue dye reduction test (MBRT) in which reduction of methylene blue to a colorless compound by reductase enzymes in the cell membrane is recorded [2]. However, unlike the other methods described above, assessments reliant on metabolism do not detect transiently metabolically inactive cells such as persister cells responsible for the antibiotic tolerance observed in a broad range of microbial species. Antibiotic tolerance, which is distinct from antibiotic resistance, is defined as the ability of a fraction of an antibiotic-susceptible bacterial population “persisters” to survive exposure to normally lethal concentrations of bactericidal antibiotics [4-7]. Persister cells are an important and growing area of research owing to their high clinical and environmental relevance [4-7].

Here, we combined the methodology of quantitative qPCR calculations with a qualitative method of bacterial growth determination described by De Groot *et al*. [8] to develop an improved quantitative method, termed the Start of Growth Time (SGT) method. This method allows researchers to detect the relative number of live bacteria within samples and is well suited for HTS studies. This method is based on the observation that the number of cells in an initial inoculum is linearly proportional to the lag phase of growth before cultures reach a threshold optical density [8]. We describe here several practical high throughput applications of the SGT method, including assessment of the efficacy of various compounds on the formation of antibiotic tolerant persister cells.

## Methods

### Bacterial growth and conditions

All compounds used in this work were obtained from Sigma Aldrich. *Pseudomonas aeruginosa* strain PA14 [9] and isogenic mutants, *Acinetobacter baumanii* and *Escherichia coli* DH5α were obtained from our laboratory stock collection. Bacteria were grown overnight in Luria Bertani (LB) medium at 37°C, diluted 1:100, and re-grown in LB or M63 (KH_2_PO_4_ [100 mM], (NH_4_)_2_SO_4_ [15 mM], FeSO_4_·7H_2_O [1.7 μM], MgSO_4_·7H_2_O [1 mM], Glucose [0.2%]) media. *P*. *aeruginosa* PA14 cells were grown to mid-logarithmic phase in the absence or presence of: (i) AA or 3-AA at a concentration (0.75 mM) that does not affect growth rate; and (ii) gentamicin (1.5 mg/L) or ciprofloxacin (0.04 mg/L) at a sub MIC concentration that also does not affect growth rate.

For CFU counts, cells were diluted serially in LB medium and plated on LB agar plates which were incubated for 24 h at 37°C. For the SGT method, an aliquot of cells was diluted 1:500 in fresh LB to serve as a “normalizer” and the antibiotic meropenem was added to the rest of the cultures (“treated”) to a final concentration of 100× MIC (i.e. 10 mg/L). Cells were incubated with the antibiotic at 37°C for an additional 24 h, and then diluted 1:500 in LB to rid the culture of the antibiotic effect. The growth kinetics of both normalizers and treated cells were recorded using an automated 96-well plate reader (Sunrise Tecan, Switzerland) at 37°C with 10 s of circular shaking every 15 min, followed by 10 s of settling at which time OD_600nm_ was detected. The SGT for each sample was determined as the time when the OD_600nm_ of the sample reached a threshold of 0.15 - 0.2. The relative size of the antibiotic tolerant persister subpopulation for each mutant’s culture was calculated as the log_2_ fold of change (-∆∆*SGT*) between the samples normalized to that of PA14.

### ∆∆*SGT* calculation

We applied the methodology to calculate the ∆∆*ct* for quantitative polymerase chain reaction experiments (qPCR) [10,11] by determining ∆∆*SGT* values of samples compared to a calibrator. First, a ∆*SGT* value was calculated for each sample according to the following equation: *ΔSGT* = (*SGT*_Treated_ − *SGT*_Normalizer_) where the SGT of untreated normalizer cells was subtracted from the SGT of treated cells. Second, a ∆∆*SGT* value was calculated by subtracting the ∆*SGT* of the reference strain or condition (“calibrator”) from that of the sample: *ΔΔSGT* = (*ΔSGT*_Sample_ − *ΔSGT*_Calibrator_). Fold change between the sample and the calibrator was calculated as: *F* = 2^−*ΔΔSGT*^. Results are presented as log_2_ fold changes: -∆∆*SGT*.

## Results and discussion

### Assessment of live bacteria cell number in a high throughput setting

**Figure 1:**
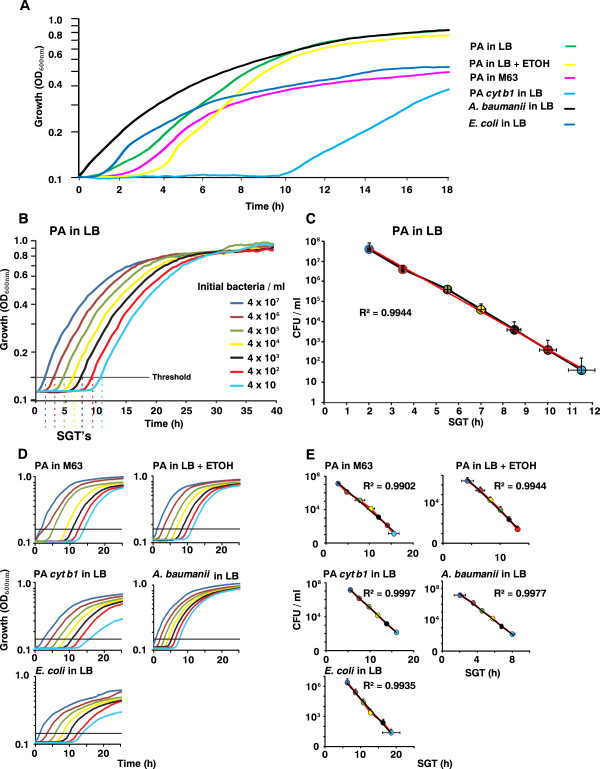
**SGT values are proportional to the initial inoculum.** The linearity of SGT method was assessed in various strains and conditions. (**A**) Growth curves of the wild-type *P*. *aeruginosa* strain PA14 (PA) grown in LB (Green), LB + 3% Ethanol (Yellow) and in the defined medium M63 (Pink); PA14 isogenic mutant derivative *cyt b1* (light blue); and wild-type strains *A*. *baumanii* (black) and *E*. *coli* DH5α (dark blue). (**B**) The time when the growth curves crossed the threshold (OD_600nm_ = 0.15 - 0.2) is defined as the SGT. *P*. *aeruginosa* PA14 cells were grown to OD_600nm_ = 2.0, when the concentration of cells was 4.07 x 10^9^ ± 7.02 x 10^8^ cells/mL according to CFU counts. The cells were diluted serially 1:10 in a 96-well plate reader to ODs below the detection threshold of the spectrophotometer, after which their growth kinetics was recorded and also determined at 18 h by CFU counts. Each growth curve is the average of at least 3 repeats. (**C**) Plots of SGT values versus bacterial concentrations detected by CFU count reveal linear correlation in all cases (R^2^ >0.99). Colors of the circles correspond to inoculum concentrations. The linear regression curve is shown in red. (**D** - **E**) Growth curves and plots of SGT values versus bacterial concentrations detected by CFU count for the additional conditions and strains.

The SGT method is based on the time that a growing bacterial cell culture takes to reach spectrophotometrically detectable levels being proportional to the starting bacterial inoculum [8]. This approach allows live bacteria within a culture to be quantified (Figure 1). The SGT of each sample is defined as the time required by the culture to reach an OD_600nm_ threshold that is set slightly above the detectable background at the start of the logarithmic phase of growth, 0.15-0.2 in the present study.

As shown in Figure 1, the SGT values of bacterial cell cultures are proportional to the initial inoculum of all conditions and strains used. The SGT values of various bacterial cell cultures inoculated with various starting concentrations and grown in various conditions (Figure 1A) were determined (Figures 1B and 1D). A calibration curve was generated by plotting the SGT values against the corresponding starting inoculum values, which were assessed by CFU counts on plates (Figures 1C and 1E). As shown, we observed a linear correlation between the SGT values and the number of CFUs within the starting inocula (R^2^ > 0.99). Using these calibration curves, it was possible to assess the concentration of live cells within a given sample without plating regardless of its growth condition.

Figures 1B and 1D show that the SGT values were obtained within 2 h for 4 × 10^7^ ± 7 × 10^6^ CFU/mL and within 11.5 h when the starting concentration of cells was as low as 51 ± 42 CFU/mL. These processing times are much shorter than the ≥24 h period needed to obtain CFU counts. Furthermore, it is noteworthy that the SGT method was sensitive enough to detect spectrophotometrically live cell number differences between 40 and 400 bacteria. Taken together these results show that the SGT method can provide sensitive, accurate, robust and rapid estimation for bacteria cell numbers in a manner that is suitable for use in a high throughput setting.

### Example 1: Assessment of antibiotic bactericidal activity

The SGT method can be used to evaluate the relative bactericidal activities of antibiotics or other compounds that impact bacterial growth. To this end, we applied the methodology to calculate the ∆∆*ct* for qPCR [10,11] by determining ∆∆*SGT* values of samples compared to a calibrator as described in Methods section.

**Figure 2:**
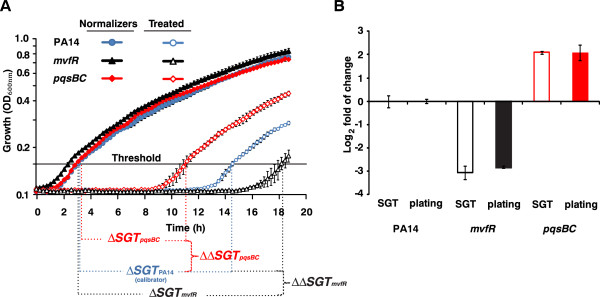
**Example of SGT method use**: **assessment of the relative bactericidal activity of meropenem on various *P***. ***aeruginosa *isogenic mutants.** (**A**) Wild-type PA14 (blue) and its isogenic mutant derivatives *mvfR* (black) and *pqsBC* (red) were grown to mid-logarithmic phase before being subjected to a 24 h treatment with meropenem (10 mg/L) at 37°C (no meropenem added to normalizers). Following 1:500 dilution, the growth kinetics of normalizers and treated samples were recorded. Employing an OD_600nm_ = 0.15, ∆*SGT* values were calculated as the difference between treated and normalizer SGTs. ∆∆*SGT* values were calculated as the difference of between ∆*SGT*s of the mutants to that of wild-type PA14, which served as the calibrator. (**B**) For the SGT method, log_2_ fold of change was calculated as -∆∆*SGT* (empty bars). For CFU counting, normalizers and treated cells were serially diluted and plated. For comparison purposes, CFU count results are also presented as log_2_ fold of change (filled bars). The differences between the values obtained by the two methods did not differ significantly (p > 0.1).

The killing efficacy of the antibiotic meropenem on *P*. *aeruginosa* cells was compared to that on two of its isogenic mutants, *mvfR* and *pqsBC* (Figure 2A). The *mvfR* mutant harbors a mutation in the global virulence-related quorum sensing regulator MvfR, while *pqsBC*, MvfR regulated genes, encode the enzymes PqsB and PqsC which are required for the synthesis of 4-hydroxy-2-alkylquinolines (HAQ) [12-16]. In this example, the meropenem treated cells were defined as Treated and cells not exposed to meropenem were used as Normalizers. Wild-type PA14 strain cultures served as the reference calibrator cultures and the two mutants were processed as samples. After meropenem treatment, the growth kinetics of the normalizers and treated cells were recorded as described in the Methods. With an OD_600nm_ threshold of 0.15, ∆*SGT* values were calculated as: *ΔSGT* = (*SGT*_Treated (meropenem)_ − *SGT*_Normalizer (untreated)_) for each sample. The relative size of the antibiotic tolerant persister subpopulation in each mutant’s culture was calculated as the log_2_ fold of change (−∆∆*SGT*) where: *ΔΔSGT* = (*ΔSGT*_Sample (*mvfRor pqsBC*)_) − *ΔSGT*_Calibrator (PA14)_).

The *mvfR* mutant cells had a lower number (log_2_ fold change of −3.0 ± 0.29) and *pqsBC* mutant cells had a higher number (log_2_ fold change of 2.1 ± 0.07) of surviving cells than wild-type PA14 cells (Figure 2B). There was a strong concordance between these SGT data and CFU data obtained in parallel (p > 0.1), providing validation of the SGT method (Figure 2B).

### Example 2: Screening for a compound’s effect on the size of an antibiotic tolerant subpopulation

**Figure 3:**
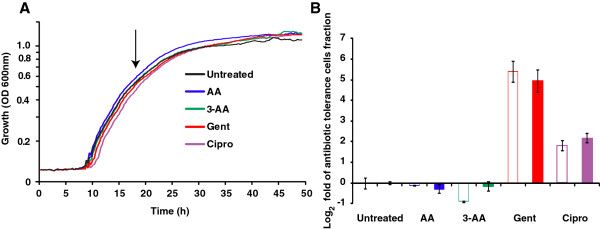
**Example of SGT method use**: **assessment of the relative efficacy of compounds on the size of the persister cell fraction using the SGT method.** (**A**) PA14 cells were grown to the mid-logarithmic stage (arrow) in the absence or presence of AA (0.75 mM), 3-AA (0.75 mM), gentamicin (Gent, 1.5 mg/L) and ciprofloxacin (Cipro, 0.04 mg/L). Meropenem was applied as in Figure 2. (**B**) A comparison of survival fraction sizes obtained by SGT (empty bars) and CFU counting (filled bars) methods, presented as log_2_ fold change. SGT values were determined using a threshold OD_600nm_ = 0.15. ∆*SGT* values were calculated as the difference between the SGT values of meropenem treated and untreated cultures and ∆∆*SGT* values as the difference between compound-treated cultures and the untreated calibrator. The SGT and CFU count data were not significantly different (p > 0.05).

Another practical application of the SGT method is screening for compounds that affect the formation of antibiotic tolerant cells. To demonstrate this application, we examined the effects of four compounds on the size of persister subpopulations in PA14 cultures exposed to a lethal dose of meropenem (10 mg/L). Specifically, the compounds used were: (*i*) the HAQ precursor anthranilic acid (AA) [16]; (*ii*) the AA analog 3-AA; and the two antibiotics (*iii*) gentamicin and (*iv*) ciprofloxacin (Figure 3A).

*P*. *aeruginosa* PA14 cells were grown to mid-logarithmic phase in the absence or presence of AA, 3-AA, gentamicin or ciprofloxacin at a concentration that does not affect growth rate (Figure 3A). After meropenem addition, the cells were incubated for 24 h and the relative size of the surviving cell subpopulation was determined using the SGT and CFU count methods in parallel, as described above. Both methods showed, with no significant difference between them (p > 0.1), that gentamicin and ciprofloxacin increased the surviving, antibiotic tolerant cell subpopulation by ~ 5 and 2 log_2_ fold respectively relative to no compound, while AA and 3-AA did not affect cell survival. Importantly, this assay can be scaled up to simultaneously evaluate the efficacy of triplicates of 32 compounds in 96-well plates or triplicates of 128 compounds in 384-well plates.

## Conclusions

The SGT method is a reproducible, accurate, and rapid way to estimate the number of living bacteria cells present in a liquid culture. It is not laborious and can be performed without any specialized training or equipment beyond a basic automated plate reader. Unlike CFU data, SGT values cannot be skewed by clumps of bacteria. Like conventional OD_600nm_ plate reading, SGT detects only live bacteria and simultaneously provides additional information on the nature of the growth state, such as cell doubling time and time to enter the stationary phase. However, SGT is much more sensitive than conventional OD_600nm_ reading as it can detect concentrations of bacteria as low as ~10 bacteria/mL. The SGT method can be used for a diversity of applications, including HTS of compounds and conditions that affect bacterial viability and studies of antibiotic tolerance and persister cell formation.

The SGT method does have some limitations that should be noted. Firstly, unlike CFU counting, the SGT method requires that calibrator and sample cultures be grown in the same conditions with similar doubling times, as it assumes that the time needed for a growing bacterial culture to reach the threshold is proportional to the concentration of the initial inoculum. Secondly, in conditions that affect the lag phase of growth, SGT values must be taken with caution. For example, cells grown in minimal media could falsely mimic low inocula in comparison to same concentration cells grown in rich media. Third, in the case of persister cells assessment, changes or differences in the “awakening” kinetics of these cells could cause a potential bias since rapid awakening cells could be interpreted falsely as high number of cells. In such cases, as it is used in oligonucleotide pair assessment in qPCR, a single calibration curve of SGT versus CFU would be needed to determine the linearity of the SGT values. Finally, when performing HTS using SGT, validation of hits using the conventional CFU plating method would be prudent.

## Competing interests

The authors declare that they have no competing interests.

## Authors’ contributions

RH, YQ, and LGR designed the SGT method. RH and YQ and DM carried out the experiments. RH, YQ, and LGR wrote the manuscript. All authors read and approved the final manuscript.
